# The Impact of Cochlear Implantation in Pediatric Patients on Quality of Life: A Systematic Review and Meta-Analysis

**DOI:** 10.1097/ONO.0000000000000068

**Published:** 2025-04-17

**Authors:** Corinne A. Pittman, Nicole A. Derdzakyan, Jeremiah Olabosipo, Andrea Warner-Czyz, Michael Hoa

**Affiliations:** 1 Department of Otolaryngology—Head and Neck Surgery, MedStar Georgetown University Hospital, Washington DC; 2 George Washington University School of Medicine and Health Sciences, Washington DC; 3 Georgetown University School of Medicine, Washington, DC; 4 Department of Speech, Language, and Hearing, The University of Texas at Dallas, Richardson, TX; 5 Dallas Cochlear Implant Program, Callier Center for Communication Disorders, University of Texas at Dallas, Dallas, TX

**Keywords:** Cochlear Implantation, Pediatric Patients, Quality of Life Measurement

## Abstract

**Objective:**

To evaluate the hearing-related quality of life (HR-QoL) instruments utilized to assess pediatric cochlear implant (CI) users and determine which quality of life domains are most relatable to each stage of childhood development.

**Databases reviewed:**

PubMed, OVID Medline, Embase.

**Methods:**

Our systematic review included a search of the PubMed, OVID Medline, and Embase databases using relevant MeSH terminology. Inclusion criteria captured the following: 1) pediatric CI users, 2) QoL measurement outcomes, 3) written in the English language, and 4) numerical data of survey scores readily available. Our study was adherent to the Meta-analysis Of Observational Studies in Epidemiology reporting guidelines.

**Results:**

Among 1597 studies screened, 20 met the inclusion criteria. Among 1369 pediatric CI patients surveyed, nearly one-third of the studies administered a pediatric and parental version of the generic KINDL QoL questionnaire. Both children and adolescents with CI scored similarly in the generic HR-QoL and in the specialized Peds QoL questionnaire (CI) (scores displayed in mean ± SD; children: 67.11± 12.6; adolescents: 69.40± 12.42). CI users in both age groups scored lower than their age-matched normal hearing peers (NHP) (79.11 ± 11.63) and to their parents (78.19 ± 10.18) on both the generic and CI-specific QoL questionnaires. The highest scores across studies among CI users were observed under the physical and psychosocial well-being domains.

**Conclusions and Relevance:**

Children and adolescents with CI experience similar physical and psychosocial functioning QoL aspects, though lower than their NHP. Disagreement was observed between most pediatric and parental QoL reports among children and adolescents at QoL assessment, suggesting parents may not be reliable reporters on their child’s overall QoL. These data provide a basis for future discussions aimed at designing standardized HR-QoL measures for pediatric CI users.

Quality of life (QoL), a fundamental metric of well-being, is significantly influenced by a spectrum of factors that evolve across the developmental stages of childhood (1). This period is distinguished by critical milestones in physical, social, cognitive, and emotional domains. One crucial domain, the physical domain, is the ability to engage in everyday activities without significant pain or fatigue, which reflects a child’s overall physical health status. Another key aspect centers on social well-being, or the capacity to interact with peers, form friendships, and maintain positive relationships, contributing to social identity and emotional development. Academic performance, participation in school activities, and the presence of a supportive school environment are also vital, as they determine how well a child adapts to and thrives in educational settings (ie, the cognitive domain). Additionally, the ability to manage emotions, maintain self-esteem, and handle stressors effectively is essential, as it indicates a child’s emotional health and resilience.

Early childhood is characterized by a series of developmental “firsts”—encompassing the acquisition of basic literacy skills, advanced language development, and initial peer interactions. As children progress into adolescence, there is a notable transformation in body image, the establishment of more complex peer relationships, and the commencement of identity formation. Upon entering adolescence, individuals endeavor to achieve emotional stability, enhance their coping mechanisms, and foster a strong work ethic (2–4). The presence of strong social frameworks, proficient communication skills, and supportive familial and peer relationships are imperative for effective school integration, fostering peer interactions, and ultimately improving overall QoL (2–4).

The integrity of developmental stages and QoL in a neurotypical population may not hold in children with disabilities. For example, children diagnosed as deaf and hard of hearing (DHH) may not have adequate auditory access to sound, which influences how they interact with their real-world environment. The provision of hearing technology such as hearing aids (HAs) and surgically implanted electronic devices, cochlear implants (CIs), can provide access to sound, but differences related to the integration of this technology into their person highlight the multifaceted impact of CIs on their physical, social, academic, and emotional well-being (5–9).

Pediatric CI users face challenges in many QoL domains, particularly peer acceptance, social and emotional adjustment, and self-esteem. Historically, social competence has been identified as an area of concern for children who are DHH because, as a group, they experience more problems with peers, report feeling lonelier or being ignored, and exhibit more difficulty making and maintaining friendships than do peers with normal hearing (NH) (7–9). Challenges faced by children who are DHH encompass a spectrum of social difficulties, from the inability to form genuine friendships to experiencing peer victimization. Peer victimization, defined as unwanted aggressive behavior by an individual or group of youths, is characterized by a power imbalance, albeit actual or perceived, and the likelihood of repeated occurrences (10). This phenomenon highlights critical areas of concern within the social dynamics encountered by children with hearing impairments, necessitating targeted interventions to promote inclusivity and prevent bullying.

Compared with their NH peers, pediatric CI users tend to have less developed social skills and fewer close friendships and often feel discriminated against at rates much higher than their NH counterparts (11–13). Many children and adolescents who are DHH and use HAs or CIs experience higher rates of victimization than the general population (50% vs 28%), as well as different types of victimization, with teasing, rumors, and exclusion being the most common (14). In fact, a 2018 study by Warner-Czyz et al (14), showed that adolescents who are DHH reported nearly twice the rate of peer victimization and 5 times the rate of social exclusion, compared with the general population, however, a difference was not noted between HA and CI users. Some research studies report poorer overall QoL in children who are DHH compared with their peers with NH (15,16). Other studies suggest no differences between the 2 groups based on auditory status; however, similarities in overall QoL do not preclude domain-specific differences in physical, mental, or social well-being (17,18). In another study by Duarte et al (13), children with CIs reported lower satisfaction with daily interactions and overall QoL compared with their hearing counterparts. These findings underscore the imperative need for tailored social support and intervention strategies aimed at enhancing the social integration and well-being of pediatric CI users, thereby bridging the gap in QoL experienced between them and their NH peers. The differences in social experiences and overall QoL between children who are DHH and their NH peers illustrate a complex landscape of needs, necessitating specialized support and leading to a focused examination of health-related QoL (HR-QoL) assessments.

HR-QoL assessments are essential for understanding pediatric CI users’ support needs and resources to optimize their long-term QoL (19,20). Various tools exist to assess HR-QoL in children, and some can be administered to both patients and their parents as proxies (21). However, parental perspectives may differ from those of CI users themselves, as both their experiences and interactive patterns differ (22). That is, parents may impose their own concerns onto their perception of their child’s QoL (23–26) or may not understand accurately subjective aspects of QoL that depend on internal or emotional aspects (eg, self-esteem, emotional well-being, and social skills) (9,27–29).

Generic HR-QoL tools apply to diverse patient populations, while hearing-specific tools are tailored to unique subpopulations, such as CI patients (30). Lin and Niparko (31) found that the conclusions from studies with generic HR-QoL tools had less bias among the results found. Generic HR-QoL tools include a broad variety of questions pertaining to the child’s QoL, allowing for the study of multidimensional aspects impacting their experiences (32). Hearing-specific tools may not always capture the multidimensional needs of pediatric CI users accurately, and single-scale assessments might fail to reflect their complex experiences (30–33). Therefore, there is a need for a more focused tool that caters to pediatric CI users’ unique needs at different developmental stages while considering sociodemographic factors (33).

One tool that may serve as a model for the multidimensional, developmental approach needed to address pediatric QoL is the QoL-CI survey developed by Cejas and colleagues (34). The researchers deduced that both auditory domains, such as communication, and social domains, such as patient independence, were necessary to assess CI benefit to QoL (34). This and other tools may share common domains, but consensus on which ones are essential for assessment currently remains elusive (Tables 1 and 2). The absence of a universally accepted measure underscores the need for a standardized approach to assess the QoL in pediatric CI patients accurately. This systematic review and meta-analysis aims to evaluate the current HR-QoL measurements used for pediatric CI patients to identify standard features and relevant domains. By reaching a consensus within the otolaryngology community on the most critical domains, a comprehensive HR-QoL measurement tool can be developed to accurately reflect the unique needs of pediatric CI patients. This will facilitate standardized, multidimensional assessments to enhance patient care and support.

**TABLE 1.** Summary of domains in quality of life instruments

QoL instrument	Generic or specific?	Respondent	Age	Emotional functioning	Family	Feelings	Physical	Psychological	School	Self-esteem	Social	Friends
KINDL^R^	Generic	Children	3 years+				x	x	x	x	x	x
CHIP-CE	Generic	Children	6–17 years									
KIDSCREEN-27	Generic	Children	8–18 years				x	x	x			
PedsQL	Generic	Children	5–18 years	x			x		x		x	
KIDSCREEN-52	Generic	Children	8–18 years	x			x	x	x		x	x
HEAR-QL-26	Specific	Children	7–12 years			x						
HEAR-QL-28	Specific	Children	7–12 years			x			x		x	
NCIQ	Specific	Adults	18–70 years				x	x			x	

**TABLE 2.** Additional domains in quality of life instruments

QoL instrument	Autonomy and parenting	Autonomy	Parent relation and home life	Peers and social support	Self perception	Satisfaction	Comfort	Financial resources	Achievement	Risk avoidance	Resilience	Activities	Environment	Hearing
CHIP-CE						x	x		x	x	x			
KIDSCREEN-27	x			x										
KIDSCREEN-52		x	x		x			x						
HEAR-QL-26												x	x	
HEAR-QL-28														x

## METHODS

### Eligibility Criteria

Three independent reviewers completed an eligibility assessment. The inclusion criteria were that the study must be geared toward the HR-QoL of pediatric patients (younger than the age of 18 years) who use at least one CI. Studies must have administered HR-QoL questionnaires to the patients or parent-proxy versions to the patient’s caregivers. Studies that determined the HR-QoL of adult patients who had received a CI as a child were excluded. Additionally, studies that administered HR-QoL surveys exclusively to the caregivers of CI patients to gauge how they interpret their child’s QoL with a CI were also excluded. All studies lacking quantitative results for each survey dimension in mean and standard deviation format were excluded from the meta-analysis. All peer-reviewed literature on retrospective design pertaining to HR-QoL of pediatric CI patients was included.

### Search Strategy

Searches were completed in the PubMed, OVID, Embase, and Medline databases. Search results included all of those through June 20, 2023. The first search included the terms “quality of life” and “pediatric cochlear implant.” The second search was more advanced, which allowed a combination of multiple search criteria through the following 4 categories: “cochlear implant,” “pediatric patient; kid; child,” “quality of life; QoL; well-being,” “measure; instrument; assessment.” The searches were completed manually.

### Data Extraction

All articles that were collected from the searches were uploaded to the Rayyan software, where eligibility assessments were conducted by each independent reviewer manually. An automation tool was used only initially to remove any duplicated studies. Further duplicate studies, which were found by the reviewers were removed. From each eligible article, the QoL assessment was administered, the survey dimensions were included in that assessment, and the mean and standard deviation of survey responses were extracted to be included in the meta-analysis. One article included medians and ranges, from which the mean and standard deviation were calculated according to the non-normalized data formulas included in Hozo et al. (35). If the article provided responses from control populations, such as participants with NH or participants who are DHH without CI, these responses were also included in the meta-analysis. Finally, if caregivers were also administered a QoL assessment separate from the pediatric patient, their survey results were removed from the meta-analysis to focus solely on the outcomes of the pediatric CI user.

### Survey Instruments

Generic HR-QoL instruments included for analyses were the KINDL^R^, PedsQL, and KIDSCREEN-27. The KINDL^R^ has the following 3 versions that are administered to children based on their age: Kiddy (4–6 years), Kid (7–13 years), and Kiddo (14–17 years). The generic HR-QoL instruments broadly divide QoL into 6 domains: physical well-being, psychosocial well-being, self-esteem, family, friends, and school. Responses to the questionnaires are reported as “never” (1), “seldom” (2), “sometimes” (3), “often” (4), and “all the time” (5) by the children. Then, the study authors transformed scores to a 100-point Likert scale where 1 = 100, 2 = 75, 3 = 50, 4 = 25, and 5 = 0. A higher numerical value correlates to a higher reported QoL.

Results of the PedsQL and the KIDSCREEN-27 surveys could not be included in the meta-analysis because each survey was only administered in a single study. As a result, quantitative data from these survey instruments could not be compared across multiple studies, preventing a comprehensive comparative analysis. Three studies used the Generic KINDL^R^ survey instrument. Significant heterogeneity between studies was identified for each of the above domains. The psychometric properties of the KINDL^R^, KIDSCREEN-27, and PedsQL instruments are well-established and reliable (36–38).

This study utilized the Kid-KINDL^R^ to understand the perception of older children and the Kiddo-KINDL^R^ to evaluate adolescents. The Kid-KINDL^R^ was administered to child participants aged 8–11 years old, and the Kiddo-KINDL^R^ to adolescent participants aged 12–16 years old. The HEAR-QL-26 and -28, and CI-specific HR-QoL instruments used by Pereira et al (39) fit the category of hearing-specific HR-QoL measures assessed. These survey instruments differ from the generic HR-QoL measures in that they assess specific instances in which individuals who are DHH may experience more difficulty distinguishing sounds, such as with hearing in environments with significant ambient noise. The HEAR-QL-26 was administered to child participants aged 7–12 years old, HEAR-QL-28 to adolescent participants aged 13–18, and the CI-specific HR-QoL module was distributed to child and adolescent groups. Similar to the generic QoL questionnaires, participant responses were recorded as “never” (1), “almost never” (2), “sometimes” (3), “often” (4), or “usually” (5). Study authors transformed those responses to a Likert scale where 1 = 100, 2 = 75, 3 = 50, 4 = 25, and 5 = 0, correlating a higher score with a higher QoL. The psychometric properties of the instruments are well-established and reliable (40).

### Statistical Analysis

Due to the power considerations for a meta-analysis (41), the meta-analysis was performed on 9 dimensions (emotional functioning, family, feelings, friends, physical well-being, psychological well-being, school, self-esteem, and social). The meta-analysis was also performed on 3 different age scales: age at implantation, age at assessment, and years of CI experience.

The raw mean and variance of each outcome were considered as the effect size. Multiple independent effect size of each outcome for type of assessment and children assessment group were extracted from the same study if that study involved various types of assessment and children assessment groups.

A multilevel (3-level) meta-analysis model was performed for each outcome to account for the nonindependence at the “effect size” level of the data due to the individual effect sizes being nested within studies. Forest plots were generated as a means of visualization. The raw mean scores and 95% confidence interval for each study group were plotted for each dimension assessed, with a pooled raw mean and 95% confidence interval generated at the bottom of the Forest plot. Cook’s distance was used to explore the presence of influential outliers and was considered a possible outlier if the observations had a Cook’s distance of more than 3 times the mean (42). Heterogeneity between studies was examined using the Cochran’s Q-test (43) and the *I*^2^ index (44). Funnel plots (the standard error of a study group within a dimension vs its raw mean score) and Begg’s rank correlation test of funnel plot asymmetry were employed to assess publication bias (45). Three-level mixed-effects meta-regression was conducted to determine the degree to which children’s assessment group and/or type of assessment impacted the observed heterogeneity.

All tests are at a significance level of 0.05. All analyses were performed in RStudio (Version 2023.09.1) using the “metafor” package (46).

## RESULTS

### Search Results and Study Characteristics

**FIG. 1.**
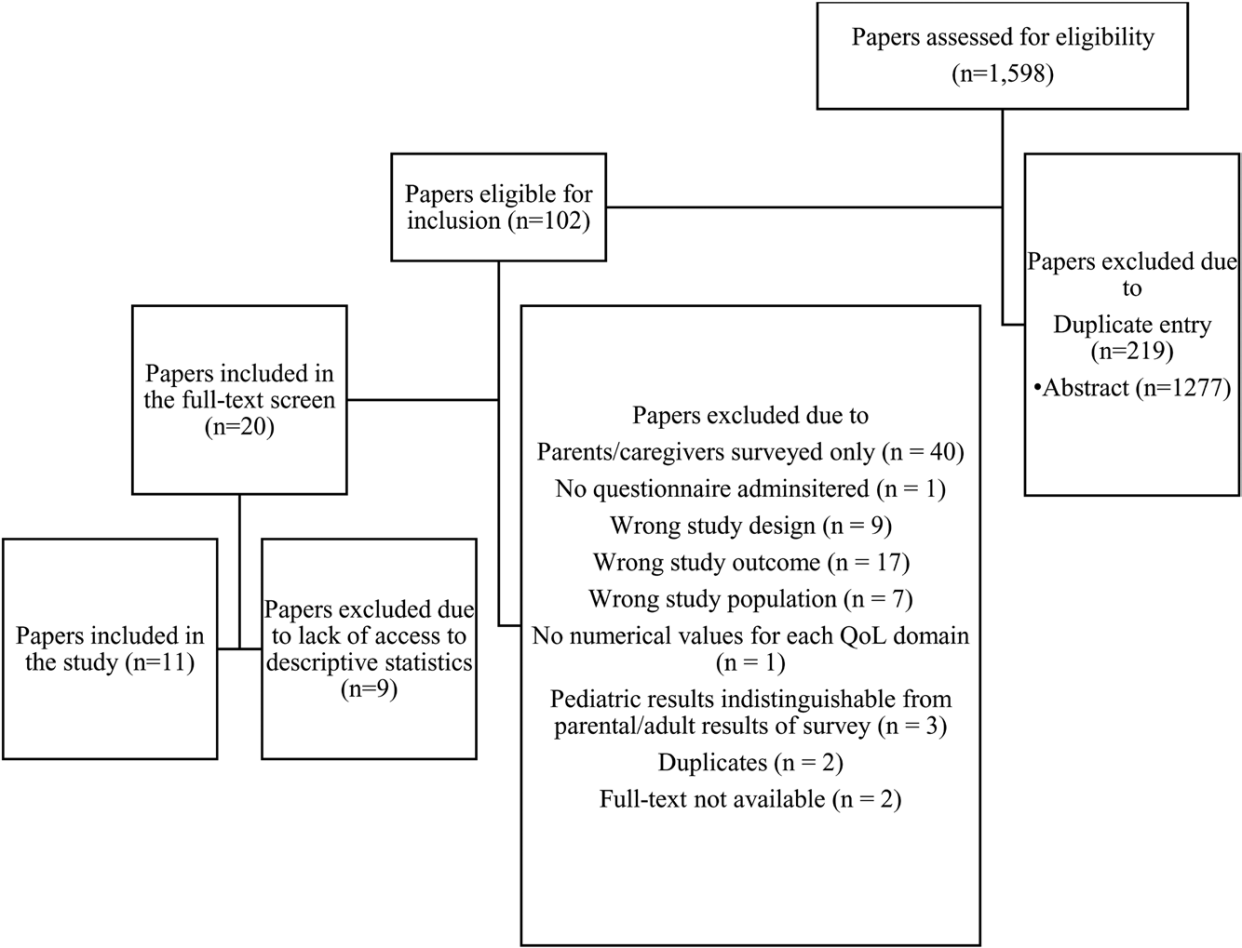
Consort diagram detailing inclusion and exclusion of papers in the systematic review.

**FIG. 2.**
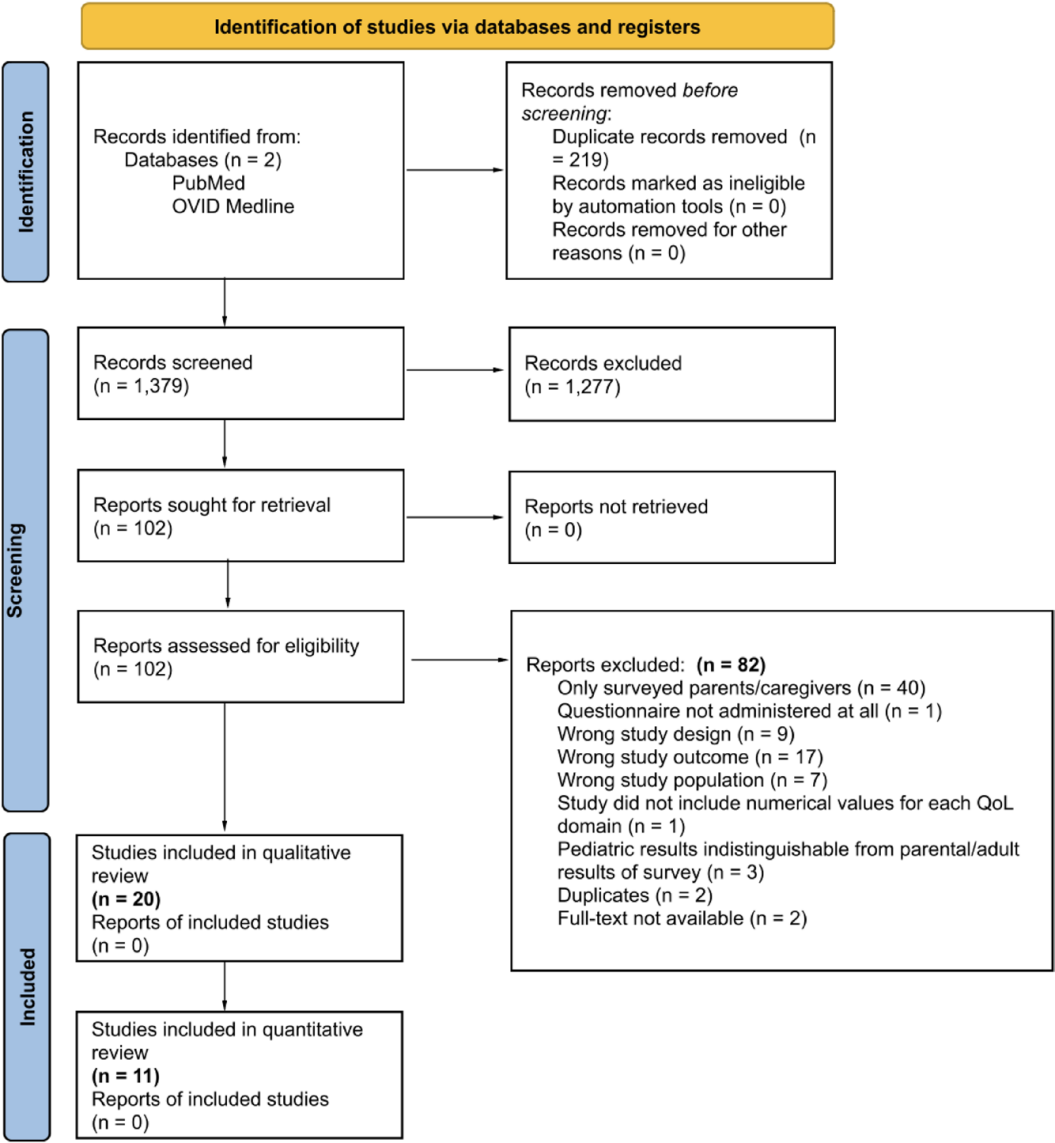
PRISMA flow diagram depicting our data collection.

Our search strategy yielded 1598 retrieved articles. The primary screening consisted of a complete review of article titles and abstracts that met inclusion criteria. All 219 duplicate studies were removed from the review. One hundred two articles that met inclusion criteria with primary review advanced to a full-text screening. During the secondary screening process, 82 articles were excluded due to an incorrect study design, such as a systematic review or single case report study, and surveys exclusively administered to obtain parent and/or caregiver responses (Figs. 1, 2). Ultimately, our search strategy yielded 20 articles that met the criteria for inclusion in our systematic review.

The majority of the 20 studies included in the systematic review used a cross-sectional design (n = 11), whereas the remaining studies employed longitudinal (n = 8) or case–control (n = 1) study designs. The sample size of the eligible 20 studies ranged from 16 to 168 subjects (M = 65.89, SD = 43.81) for a total population size of 1369 subjects. Study subjects across the included studies ranged in age from 4 to 18 years. Seven studies (n = 7) report the perspective of a parental proxy or caregiver from survey results to relay the impact of CI on their child’s QoL in addition to the child’s self-reported viewpoint. Nine studies compared QoL reports of children with CIs to age- and gender-matched peers with NH. An additional 3 studies evaluated both general and hearing-specific QoL from children with congenital deafness who used CIs and children with varying degrees of hearing amplified with HAs (47–49). While 2 of the studies demonstrated children with CIs and their parents reported a better perception of HR-QoL than children with HAs for mild, moderate to severe, and profound hearing loss (47,48), the third study reported that rather the parents of children fitted with HAs had more positive perceptions of their child’s hearing intervention and higher general expectations for their child (49). This was thought to be related to the better hearing levels for the children aided in hearing using HAs.

Although 20 articles were included from the full-text screen, only 11 were included in the meta-analysis. The meta-analysis included studies in which data were clearly expressed as a mean-standard deviation or median-range value. The 11 studies included in the meta-analysis utilized several HR-QoL metrics to evaluate QoL among pediatric CI users. For the purposes of this study, the HR-QoL instruments included were broadly divided into the following categories for analysis: generic HR-QoL instruments and hearing-specific HR-QoL instruments, described as metrics explicitly created for the purposes of the study.

#### HR-QoL Relative to the Physical Well-being Domain

**FIG. 3.**
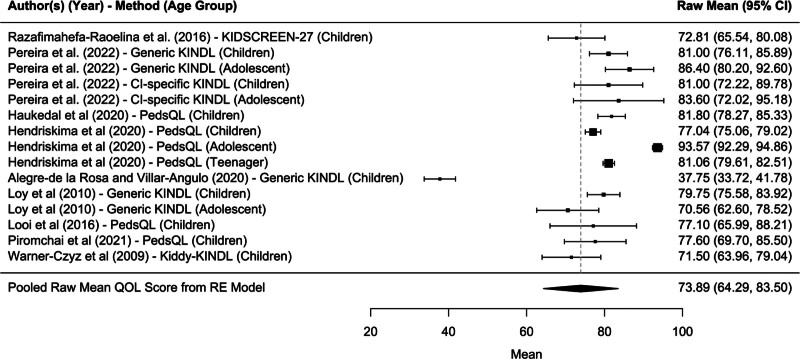
Forest plot for physical well-being domain.

The meta-analysis revealed that the raw mean score of the physical well-being domain was 73.89/100, with a 95% confidence interval ranging from 64.29 to 83.50 (*P* < 0.0001* [statistically significant result]) (Fig. 3). This mean value represents the pooled measure derived from the study participants across 9 of the 11 included articles. The statistical significance of the *P* value (<0.0001) suggests a robust association between CI and physical well-being across the 9 studies that measured this domain. The articles included in the meta-analysis of Figure 3 show variability in QoL scores, which is captured by the width of the confidence intervals. The pooled mean of 73.89 reflects the central tendency of these studies, providing a summary measure of the physical well-being domain of the HR-QoL for pediatric CI users. The narrow confidence intervals around the pooled estimate, shown in Figure 3, indicate a high degree of confidence in this summary measure. This result highlights the importance of considering physical health outcomes in this population’s overall evaluation of hearing-related QoL. The analysis excluded the remaining 2 studies, as they did not assess the physical well-being domain, precluding their inclusion in the comparative meta-analysis.

#### HR-QoL Relative to the Psychological Well-being Domain

**FIG. 4.**
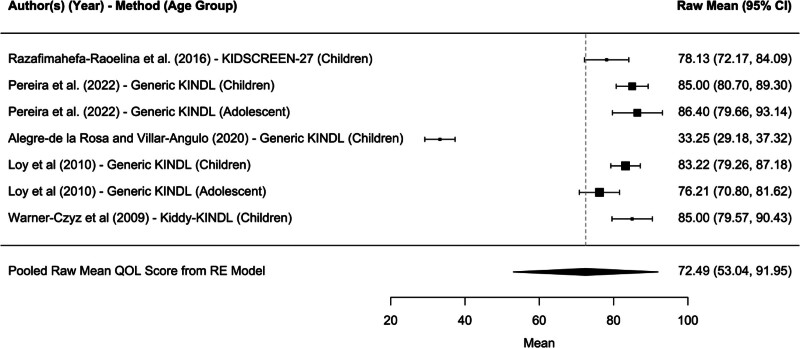
Forest plot for psychological well-being domain.

Similarly, across 5 studies, pediatric CI users rated, on average, psychological well-being a pooled mean of 72.49 (95% confidence interval, 53.04–91.95; *P* < 0.0001*) (Fig. 4). The psychological well-being scores vary significantly across the included studies. This wide confidence interval underscores the need for careful interpretation and further research to understand the factors contributing to the observed variability. For instance, the study by Alegre-de la Rosa and Villar-Angulo reports a much lower mean score (33.25) compared to others, indicating significant heterogeneity in the data (47). Several studies focusing on adolescents (i.e., Pereira et al., 2022; Warner-Czyz et al., 2009) show higher scores, suggesting better psychological well-being in older pediatric CI users compared with younger children. The results suggest that CIs generally have a positive impact on the psychological well-being of pediatric patients. However, the variability among studies highlights the need for a more standardized and comprehensive assessment tool that can consistently capture the psychological well-being of CI users across different age groups and developmental states.

#### HR-QoL Relative to the School Functioning Domain

**FIG. 5.**
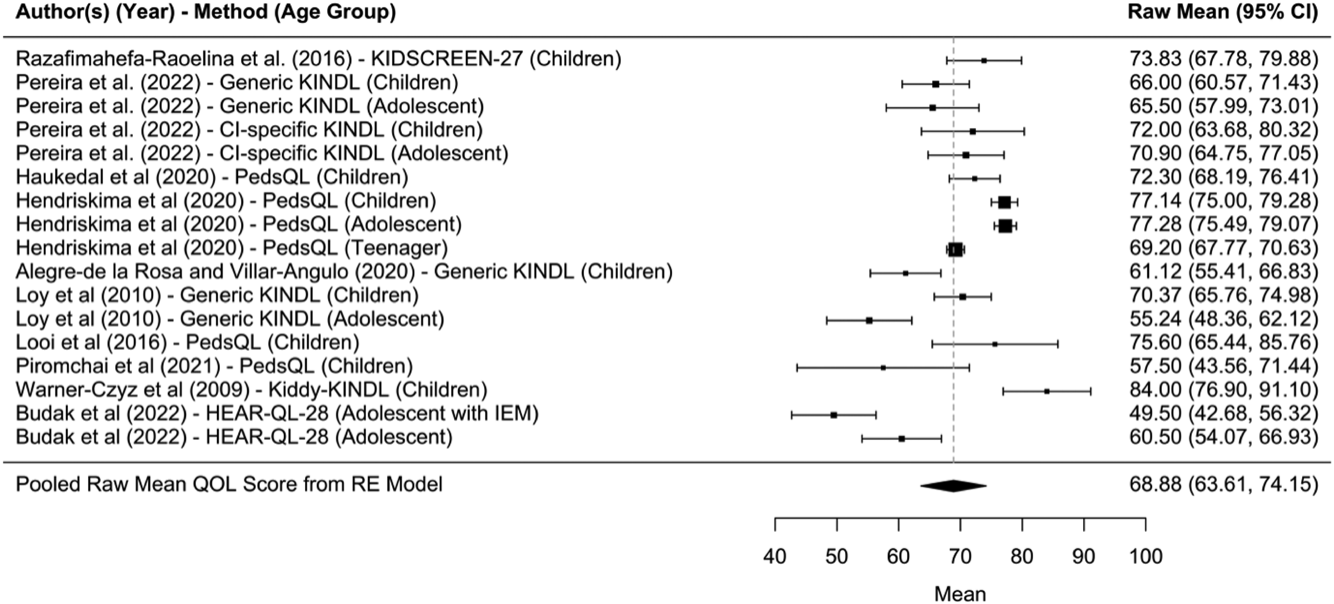
Forest plot for school functioning domain.

School functioning was scored higher among study subjects administered the Kiddy-KINDL^R^ or Generic PedsQL instruments as opposed to the Generic KINDL^R^ instrument by 19 and 9 points, respectively (19.33 [8.71–29.96]; *P* = 4.00E-04), (9.32 [2.97–15.67]; *P* = 0.004). Ten studies evaluated pediatric QoL with HR-QoL measures that included an assessment of school functioning for a pooled total mean of 68.88 (95% confidence interval, 63.61–74.15; *P* < 0.0001*) (Fig. 5). Further, adolescents with inner-ear-malformations, a participant group in the Budak et al. study (50), scored school functioning nearly 12 points lower than all other pediatric CI users within the 10 studies assessed (−11.93 [−21.61 to −2.24]; *P* = 0.0158). Further, teenagers within the Hendriskima et al. study (51) rated their ability to function in schooling 8 points lower than children from all 10 studies included in this dimensional analysis (−8.4 [−10.81 to −5.99]; *P* < 0.0001) (Table 3).

**TABLE 3.** Meta-regression estimated coefficients for school domain

Factor	Coefficient estimate	95% CI	*P* value
Type of assessment			
CI-specific HR-QoL module vs Generic KINDL^R^	6.215	(−0.143 to 12.572)	0.0554
HEAR-QL-28 vs Generic KINDL^R^	−3.236	(−13.580 to 7.108)	0.5398
Kiddy-KINDL^R^ vs Generic KINDL^R^	19.339	(8.713–29.964)	**0.0004**
KIDSCREEN-27 vs Generic KINDL^R^	9.169	(−0.787 to 19.124)	0.0711
PedsQL vs Generic KINDL^R^	9.318	(2.968–15.669)	**0.004**
Children assessment group			
Adolescent vs. children	−0.926	(−3.353 to 1.502)	0.4549
Adolescent with IEM vs. children	−11.926	(−21.607 to −2.244)	**0.0158** **<0.0001**
Teenager vs. children	−8.4	(−10.814 to −5.987)	

Bold values indicate statistically significant values.

IEM indicates inner ear malformations.

However, similar to findings of physical and psychological well-being, these factors did not explain all of the inter-study heterogeneity found in the meta-analysis (QM [df = 8] = 96.69; *P* < 0.0001), leaving significant and unexplained residual heterogeneity (QE [df = 8] = 27.12; *P* = 0.0007). In contrast, there was no statistically significant study-level characteristic to explain the heterogeneity between studies when reviewing the results of psychological well-being (Table 4). The meta-regression was unable to explain the heterogeneity (QM [df = 3] = 2.079; *P* = 0.5562) observed for psychological well-being among pediatric CI users across studies.

**TABLE 4.** Meta-regression estimated coefficients for psychological well-being domain

Factor	Coefficient estimate	95% CI	*P* value
Type of assessment			
Kiddy-KINDL^R^vs Generic KINDL^R^	17.772	(−49.086 to 84.630)	0.6024
KIDSCREEN-27 vs Generic KINDL^R^	10.902	(−56.001 to 77.805)	0.7494
Children assessment group			
Adolescent vs children	−3.455	(−8.589 to 1.680)	0.1873

#### HR-QoL Relative to the Social Functioning Domain

**FIG. 6.**
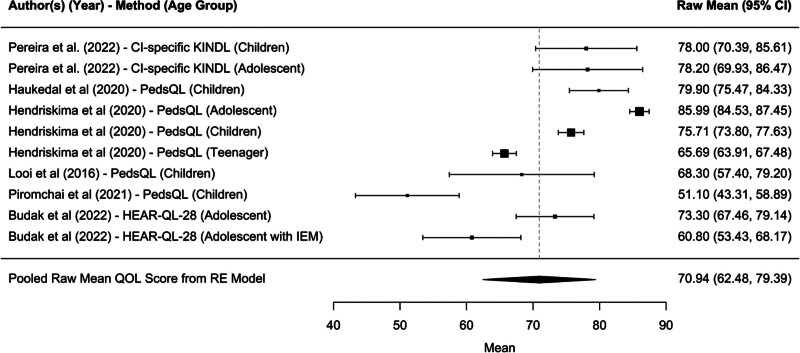
Forest plot for social domain.

The pooled mean for social functioning among 4 of the 11 studies included in the meta-analysis was 75.20, and this finding was statistically significant (95% confidence interval, 68.72–81.69; *P* < 0.0001*) (Fig. 6). This indicates a generally positive QoL outcome across pediatric CI users in social environments across the included studies. The confidence interval suggests a high level of precision in the pooled estimate, indicating that the true mean QoL score is likely within this range.

#### HR-QoL Relative to the Family Domain

Family was a common domain of the Generic-KINDL^R^ for the 3 different age groups: child (9–12 years), adolescent (12–18 years), and teenager (13–18 years). The studies that utilized these generic HR-QoL assessments had a mean score of 69 (95% confidence interval, 44.83–93.21; *P* < 0.0001), and there was significant inter-study heterogeneity (*I*^2^ = 99.16%, Q [df = 5] = 453.4975; *P* < 0.0001). Similar to the meta-regression analysis of physical well-being, adolescents, on average, rated family interaction higher than child participants, and this value was also found to be statistically significant (6.75 [1.763–11.737]; *P* < 0.0001) (Table 5). Unfortunately, the assessment group as a moderator did not have enough of an impact alone to statistically explain the overall study heterogeneity of the meta-regression for domain evaluating family interaction. Of note, we recognize the Alegre-de la Rosa and Villar-Angulo (47) study as a potential outlier of this forest plot that may have contributed to the lower mean result for family interaction. The Alegre-de la Rosa and Villar-Angulo (47) study likely did not change the statistical significance of the overall findings, however, due to the small population size.

**TABLE 5.** Meta-regression estimated coefficients for family domain

Factor	Coefficient estimate	95% CI	*P* value
Type of assessment			
Kiddy-KINDL^R^ vs Generic KINDL^R^	26.125	(−30.599 to 82.849)	0.3667
Children assessment group			
Adolescent vs children	6.75	(1.763–11.737)	**<0.0001**

Bold value indicates statistically significant values.

On assessment of potential publication biases by Begg’s rank correlation test of the physical and psychological well-being, school functioning, and emotional functioning domains, the funnel plot did not indicate any significant asymmetry (Supplemental Figures 1–4, http://links.lww.com/ONO/A35). This suggests an absence of publication biases among the study cohort analyzed, for each of these domains.

### Hearing-Specific HR-QoL Instruments

Three studies administered the hearing-specific HR-QoL instruments to pediatric CI users. Unlike the other hearing-specific HR-QoL instruments, the HEAR-QL-26 created for evaluation by children, and HEAR-QL-28 administered to adolescents include “feelings” as a specific domain. The Budak et al (50) study further varies from the other studies in that children and adolescents with and without a diagnosed inner-ear malformation completed the hearing-specific HR-QoL instruments.

#### HR-QoL Relative to the Feelings Domain

Feelings were the only domain with an overall statistically significant explanation for the study heterogeneity. The meta-analysis revealed that the pooled raw mean of the feelings dimension was 61.90 (95% confidence interval, 58.76–65.05; *P* < 0.0001*), and there was significant heterogeneity between studies (*I*^2^ is very low, Q [df = 3] = 20.58; *P* = 0.0001) (Supplemental Figure 5, http://links.lww.com/ONO/A35). As before, the significance of the inter-study heterogeneity (*I*^2^ is very low, Q [df = 3] = 20.58; *P* = 0.0001) was further explored using meta-regression by including the type of assessment and children assessment group as moderators. Both the type of assessment and assessment group were found to be statistically significant moderators that contributed to the outcome of heterogeneity between studies (QM [df = 3] = 20.58; *P* = 0.0001) and thus left no significant or unexplained residual heterogeneity. The overall scores of the feelings component of the HEAR-QL-28 health instrument were 16 points lower than those of the HEAR-QL-26 health instrument (−16.6 [−26.33 to −6.87]; *P* = 0.0008) (Table 6). Despite the finding of overall scoring between health instruments, adolescents without inner-ear malformations rated the feelings category just over 12 points higher than children without inner-ear malformations, thus experiencing a higher QoL (12.6 [3.43–21.77]; *P* = 0.0071). Further, study participants with an inner-ear malformation scored the feelings domain nearly 17 points lower than subjects without an inner-ear malformation (16.9 [−25.98 to −7.83]; *P* = 0.0003).

**TABLE 6.** Meta-regression estimated coefficients for feelings domain

Factor	Coefficient estimate	95% CI	*P* value
Type of assessment			
HEAR-QL-28 vs. HEAR-QL-26	−16.6	(−26.328 to −6.872)	**0.0008**
Children assessment group			
Adolescent vs. children	12.6	(3.432–21.768)	**0.0071**
Children with IEM vs. children	−16.9	(−25.975 to −7.825)	**0.0003**

Bold values indicate statistically significant values.

#### HR-QoL Relative to the Friend’s Domain

The forest plot of the friends domain demonstrates a pooled raw mean of 77.25 (95% confidence interval, 70.57–83.94; *P* < 0.0001*). Consistent with the current study findings, there was statistically significant study heterogeneity (*I*^2^ = 80.11%; Q [df = 7] = 45.70; *P* < 0.0001) (**Supplemental Figure 6**, http://links.lww.com/ONO/A35). The type of assessment significantly contributed to the heterogeneity found between studies. The overall scores of the CI-specific HR-QoL module assessments were lower than the Generic KINDL^R^ assessments by 19 points (−19.6 [−28.76 to −10.49]; *P* < 0.0001), similar to the difference reflected in chronological age when comparing the HEAR-QL-26 versus HEAR-QL-28 (Table 7). However, when other moderators were held constant in the model, the meta-regression did not explain all of the heterogeneity (QM [df = 3] = 19.67; *P* = 0.0002). This suggests that although the type of assessment was a significant contributor to study heterogeneity, the strength of the impact was not large enough to statistically significantly affect the overall meta-regression.

**TABLE 7.** Meta-regression estimated coefficients for friends domain

Factor	Coefficient estimate	95% CI	*P* value
Type of assessment			
CI-Specific HR-QoL module vs Generic KINDL^R^	−19.625	(−28.761 to −10.489)	**<0.0001**
Kiddy-KINDL^R^vs. Generic KINDL^R^	7.341	(−12.402 to 27.083)	0.4662
Children assessment group			
Adolescent vs children	−3.447	(−9.897 to 3.004)	0.295

Bold value indicates statistically significant values.

CI indicates cochlear implant; HR-QoL, hearing-related quality of life.

Similar to the findings of Begg’s rank correlation tests of the Generic HR-QoL common domains, none of the funnel plots demonstrated statistically significant asymmetry. This suggests an absence of publication biases among the study cohorts analyzed for each of these domains.

The meta-regression results for assessment groups comparing the social domain, self-esteem domain, and emotional functioning domain are found in Tables 8–10, respectively.

**TABLE 8.** Meta-regression estimated coefficients for social domain

Factor	Coefficient estimate	95% CI	*P* value
Type of assessment			
CI-Specific HR-QoL module vs. PedsQL	4.382	(−23.468 to 32.232)	0.7578
HEAR-QL-28 vs. PedsQL	−5.76	(−33.719 to 22.200)	0.6864
Children assessment group			
Adolescent vs children	9.871	(7.521–12.221)	**<0.0001**
Adolescent with IEM vs children	−2.629	(−12.321 to 7.064)	0.595
Teenager vs children	−10.258	(−12.854 to −7.661	**<0.0001**

Bold values indicate statistically significant values.

CI indicates cochlear implant; HR-QoL, hearing-related quality of life; IEM, inner ear malformations.

**TABLE 9.** Meta-regression estimated coefficients for self-esteem domain

Factor	Coefficient estimate	95% CI	*P* value
Type of assessment			
CI-Specific HR-QoL module vs. Generic KINDL^R^	0.463	(−8.151 to 9.077)	0.9161
Kiddy-KINDL^R^vs. Generic KINDL^R^	12.067	(6.160–17.973)	**0.0001**
Children assessment group			
Adolescent vs children	−2.68	(−8.151 to 2.792)	0.3371

Bold value indicates statistically significant values.

CI indicates cochlear implant; HR-QoL, hearing-related quality of life.

**TABLE 10.** Meta-regression estimated coefficients for emotional functioning domain

Factor	Coefficient estimate	95% CI	*P* value
Children assessment group			
Adolescent vs children	1.031	(−1.600 to 3.663)	0.4424
Teenager vs children	−6.955	(−9.483 to −4.427)	**<0.0001**

Bold value indicates statistically significant values.

#### HR-QoL Relative to the Age at Assessment

To determine whether the type of assessment or age at assessment variables account for the heterogeneity observed between studies for the common HR-QoL domains reported above, a mixed-effects meta-regression model was conducted. Adolescent subjects (aged 13–18, M = 13.30 years) on average rated physical well-being nearly 14 points higher than children (aged 7–12; M = 8.85 years), and this value was found to be statistically significant (13.93 [11.77–16.10]; *P* < 0.0001) (Table 11). However, because significant unexplained residual heterogeneity remained (QE [df = 8] = 282.08; *P* < 0.0001), the meta-regression was unable to explain all of the heterogeneity identified between studies (QM [df = 6] = 229.03; *P* < 0.0001).

**TABLE 11.** Meta-regression estimated coefficients for physical well-being domain

Factor	Coefficient estimate	95% CI	*P* value
Type of assessment			
CI-specific HR-QoL module vs Generic KINDL^R^	−0.598	(−8.565 to 7.370)	0.8831
Kiddy-KINDL^R^ vs Generic KINDL^R^	8.064	(−25.037 to 41.164)	0.633
KIDSCREEN-27 vs Generic KINDL^R^	9.374	(−23.666 to 42.413)	0.5782
PedsQL vs Generic KINDL^R^	15.482	(−6.174 to 37.137)	0.1612
Children assessment group			
Adolescent vs children	13.933	(11.768–16.098)	**<0.0001**
Teenager vs children	2.19	(−0.174 to 4.554)	0.0694

Bold value indicates statistically significant values.

CI indicates cochlear implant; HR-QoL, hearing-related quality of life.

#### HR-QoL Relative to the Age at Implantation, Age at Assessment, and Years of CI Experience

Across studies, participants were implanted at a pooled mean age of 3.2 years (95% confidence interval, 2.17–4.23; *P* < 0.0001). The eldest of the study participants, teenagers, had more positive overall HR-QoL scores than school-aged children (0.89 [0.38–1.39]; *P* = 0.0006); however, unexplained contributors to heterogeneity remained (QM [df = 2] = 12.40; *P* = 0.0020). Study participants had a pooled mean age at HR-QoL assessment of 9.5 years (9.52 years [95% confidence interval, 8.08–10.97; *P* < 0.0001]) and under 5 years of CI experience postimplantation (4.83 years [95% confidence interval, 2.56–7.10; *P* < 0.0001]). Adolescents with and without inner-ear malformations and teenagers demonstrated higher overall scores than children when controlling for the type of assessment (Table 12). The comparison between adolescents with inner-ear malformations and children was performed during the meta-regression analysis attempting to explain the inter-study heterogeneity, allowing us to compare their QoL scores as well. As both groups differ in age and inner-ear malformation status, the clinical significance of this finding may be underwhelming. The meta-regression results for children assessment groups relative to age at implantation and year of CI experience are found in Tables 13 and 14, respectively.

**TABLE 12.** Meta-regression estimated coefficients for age at assessment domain

Factor	Coefficient estimate	95% CI	*P* value
Children assessment group			
Adolescent vs children	4.649	(4.303–4.994)	**<0.0001**
Adolescent with IEM vs children	4.943	(4.333–5.552)	**<0.0001**
Children with IEM vs children	−0.457	(−1.003 to 0.088)	0.1001
Teenager vs children	9.22	(8.666–9.773)	**<0.0001**

Bold values indicate statistically significant values.

IEM indicates inner ear malformations.

**TABLE 13.** Meta-regression estimated coefficients for age at implantation domain

Factor	Coefficient estimate	95% CI	*P* value
Children assessment group			
Adolescent vs children	0.335	(−0.149 to 0.819)	0.1745
Teenager vs children	0.885	(0.380–1.389)	**0.0006**

Bold value indicates statistically significant values.

**TABLE 14.** Meta-regression estimated coefficients for years of cochlear implant experience domain

Factor	Coefficient estimate	95% CI	*P* value
Children assessment group			
Adolescent vs children	4.72	(4.002–5.438)	**<0.0001**
Teenager vs children	8.686	(7.867–9.506)	**<0.0001**

Bold values indicate statistically significant values.

## DISCUSSION

To date, existing literature investigating the long-term benefits of CI is focused on speech, language, and auditory development. However, the long-term QoL outcomes among children, adolescents, and teenagers using CIs are limited. Further, the assessment of QoL among CI recipients by a CI-specific HR-QoL instrument is nearly absent. The present systematic review and meta-analysis support the benefit of hearing-specific and CI-specific HR-QoL instruments tailored to pediatric CI users, with consideration of differences in developmental periods between children, adolescents, and teenagers, to better understand QoL outcomes. Adolescents with and without inner-ear malformations using CIs self-reported significantly better QoL outcomes across several domains, including school functioning, family interaction, and feelings, compared with children with CIs. This is consistent with prior literature documenting similar QoL scores between adolescents with CIs and children with CIs (11–13,19).

Our study identified lower HR-QoL scores among pediatric CI users during the child developmental period compared with their adolescent and teenager counterparts. These findings are somewhat dissimilar to previous literature that has shown pediatric patients with HR-QoL scores similar to, or even better than, their NH peers, particularly in the self-esteem and social well-being domains (15,27,52,53). Well-developed spoken language skills are critical to the development of effective communication (54,55). Thus, improved psychosocial well-being reflected in better HR-QoL outcomes (54,55) and lower HR-QoL scores, particularly in the self-esteem and social well-being domains, may be explained by the younger age of the child CI user. The child will have less time to adjust to hearing with a CI and develop the spoken language skills for effective communication than the older pediatric CI users. Variations in the age at assessment influenced HR-QoL outcomes across the domains of family, friends, and school (5–7,17,18,56). Receptiveness and support by family and friends are important, and it has been recommended that a team-based approach be used to help both the patient and their supporters endorse hearing with CI (9,30). Similar to previous reports, we acknowledge that the parental perspective may differ from the child’s self-reported perspective (23–25,56). Parents might subconsciously project their own insecurities about their child’s social skills and school functioning rather than accurately reflecting the child’s actual progress in these areas (23–26). For instance, parents may rate several HR-QoL domains more positively for children with perceived better language skills, as they might associate strong language abilities with overall better social integration and academic performance. This perception could lead parents or caregivers to believe their child is thriving more in social and educational environments, thus influencing their assessment of the child’s QoL. Children with CI, however, have consistently been found to score lower in the school domain on several HR-QoL instruments, including the PedsQL and KINDL^R^ (1,39,54,55,57). These findings support the reevaluation and improvement of listening environments in mainstream schools to better accommodate speech perception for children with CI (1,17,39). For younger children, the focus should be on supporting speech and language development and social integration within play and educational environments, as these domains significantly impact their QoL.

The role of separate assessment tools significantly affected responses to the feelings domain of the HEAR-QL-26 and HEAR-QL-28 measures. The direct relationship between ratings of the feelings domain was driven primarily by the adolescent and teenage groups who responded to the HEAR-QL-28, with little contribution from the youngest group of child CI users to the HEAR-QL-26. We demonstrate significantly lower responses from adolescents to the HEAR-QL-28 regarding feelings of how they looked with their CI devices, as opposed to children responding to their respective HEAR-QL-26 questionnaire. This finding may suggest that adolescents who feel more encumbered by their CI may have fewer opportunities to interact successfully with others and to practice identifying sounds (58). Conversely, children who perceive that they hear better may feel more confident in their ability to accurately hear and interpret sounds, which could contribute to higher reported QoL scores (58,59). A previous study by Warner-Czyz et al (1) similarly showed that CI users of the adolescent and teenage groups rated more negative feelings of embarrassment and frustration while wearing a CI than child CI users. Adolescents and teenagers perhaps have a heightened awareness of self-image and societal perception than the youngest CI users. It is challenging to determine whether discrepancies in HR-QoL ratings arise solely from differences in age or from variations in the QoL questionnaires themselves. Child CI users will generally be at lower levels of social and academic development than adolescent or teenage CI users, potentially limiting their ability to accurately express their self-perceptions or fully comprehend their emotions in the context of a new CI user. Furthermore, these groups may vary in several key factors, including but not limited to the type of CI device used at the time of implantation, the duration of time and experience with the device, their educational level, and their preferred communication methods. Although having fewer choices in the 26-question HEAR-QL questionnaire allows for easier collection of data from the child CI user, the lack of intermediate choices, incomplete understanding of questions, and inability to assign consistent responses may have contributed to the low reporting within the group of child CI users.

Age at implantation and duration of experience with CI were also found to affect HR-QoL outcomes. The teenage study participants implanted, on average at 3 years of age, had higher overall HR-QoL scores than their adolescent and child CI counterparts. Similarly, adolescent and teenage CI users, with an average of 5 years of experience with a CI, demonstrated higher overall scores than child CI users. Pediatric patients implanted at an earlier age or with a longer duration of CI use have reported better HR-QoL outcomes in language, social, and functional outcomes (18,60–63). Older age at CI implantation has previously been associated with lower language scores (64), and younger age at CI implantation is associated with better self-esteem outcomes (65). While our study similarly demonstrated higher self-esteem ratings among child CI users when compared with adolescent CI users, this value was not statistically significant. This finding aligns with prior studies indicating higher self-esteem ratings among child CI users compared with adolescents (17,18,58). Earlier implantation has been associated with more favorable HR-QoL outcomes, as children implanted at a younger age often develop superior speech perception and language acquisition skills (1,59,66). Additionally, longer use of CIs correlates with more positive overall HR-QoL scores. Children who can communicate effectively at home and school tend to exhibit greater self-esteem and self-reliance, likely contributing to their higher reported QoL.

These observations underscore the potential transformative impact of early identification, timely intervention, and advanced integration practices for pediatric CI users. Enhanced speech recognition and improved integration into mainstream education, as seen in recent cohorts, are likely to significantly mitigate the HR-QoL disparities previously documented. As this newer generation progresses into preadolescence and adolescence, their extended duration of device use, combined with early interventions, positions them for potentially superior outcomes in family, friends, and school domains compared with earlier cohorts. This evolving evidence suggests a pivotal shift in the landscape of pediatric cochlear implantation, highlighting the critical importance of early and sustained interventions in optimizing long-term QoL outcomes.

To date, there is one CI-specific QoL measure by Cejas et al (34) that allows the CI user to rate barriers experienced with daily interactions and benefits as a result of the CI device (67). It is important to develop CI-specific measures to improve the global dissemination and implementation of such measures to monitor patients. It is limited how much physicians can learn about their patients’ experiences and improve them if there are few measures and little clinical use of them. The utilization of these measures should be encouraged among physicians.

Differences in HR-QoL may also be limited by factors that are not included but should be considered during HR-QoL assessment, such as comorbidities, sociodemographic factors, parenting style, child and parent temperament, and family dynamics (68–70). Parents with higher levels of education often have increased health literacy and a more proactive approach to navigating the healthcare and educational systems. In fact, higher parental education has been found to affect CI outcomes and HR-QoL (23,71). For instance, they may seek out specialized auditory-verbal therapy and consistently follow recommended audiological follow-up schedules, ensuring timely adjustments to the child’s CI settings. Additionally, these parents may engage their children in more frequent reading activities and enriched language-based interactions—such as discussing new vocabulary or narrating daily routines—which can accelerate language acquisition and speech perception skills. Such positive home environments also tend to promote better self-advocacy and easier integration into mainstream educational settings, contributing to improved peer relationships and overall well-being. It may also be important to include a domain for their developmental milestones to not only better evaluate the HR-QoL of patients with disabilities but also deliver the appropriate care (59,63,72). By including these additional factors, the appropriate support systems for CI users can be integrated into daily living interaction, and any necessary rehabilitation programs can be created to accommodate the specific needs of the CI user and ultimately improve HR-QoL outcomes.

To develop a multidimensional HR-QoL measurement tool for pediatric CI users, it is critical to incorporate domains that comprehensively capture the unique challenges faced by this population. Building on findings from Cejas et al (73) the tool should include domains such as social functioning, emotional well-being, device management, and communication, as these areas are pivotal to understanding the holistic impact of CIs on children’s lives. Social functioning must address peer interactions, friendships, and inclusion in group settings, given the heightened risks of social isolation reported in CI users. Emotional functioning should evaluate mood regulation, self-esteem, and acceptance of hearing loss, particularly as children transition through adolescence. Additionally, the tool should include items assessing independence and advocacy, reflecting the developmental needs of older children and young adults as they navigate self-care and social environments. Early intervention and academic functioning are equally essential, particularly for younger children, to monitor developmental milestones and educational integration (34). Such a comprehensive, developmentally sensitive approach would enhance the ability to evaluate and address the varied experiences of pediatric CI users across the age spectrum.

Further, insights from adult literature, such as McRackan et al’s (74) CI QoL measure, highlight several critical domains that should be adapted for children. Domains including listening effort, which quantifies the cognitive and physical strain of hearing in challenging environments, and environmental awareness, which measures the ability to detect and localize everyday sounds, could provide valuable data on the real-world challenges of pediatric CI users. These areas, when tailored to the developmental stages of children, could significantly enhance the relevance and utility of the HR-QoL tool.

We acknowledge that this systematic review has limitations: the search strategy was restricted to capture studies only in the English language that have specifically used an HR-QoL instrument to evaluate children who have received a CI. However, these limitations may be minimal, given that many authors from countries where English is not the primary language still often publish in English-language journals. Further, other studies that have applied HR-QoL instruments to children with hearing loss who have not received a CI were appropriately excluded, as the conceptual basis of the HR-QoL experience is likely different in these children. This study also excluded assessment results by parental proxies as their outcomes may impact HR-QoL ratings, such that parents may positively skew ratings of their child’s communication abilities within family and friend units, school settings, social encounters, and even maturational processes in regards to self-esteem and personal feelings with the CI (1,25,33).

This paper also has strengths, including its power as a meta-analysis to broadly assess the QoL across over 13,000 pediatric CI recipients. It also pools data from both generic and hearing-specific QoL instruments to highlight the comparison of different instruments. Finally, this study emphasizes the importance of considering the subjective QoL measures from the perspective of the child or adolescent who uses a CI rather than relying on parental or teacher proxy.

To summarize, current pediatric HR-QoL measures commonly assess the patient’s psychosocial well-being, but very few directly address how patients cope with hearing loss and CI. Parent-proxy versions for these questionnaires exist to provide additional reliable and valid approaches for the evaluation of pediatric patient HR-QoL; however, they may rate children’s level of confidence with CI utilization and ease of communication higher than the pediatric CI users themselves. No assessment tool currently exists to fully encompass the HR-QoL of pediatric patients, as CI affects multiple areas in the patient’s life, accounting for the different stages of development. Further studies are needed among the otolaryngology community about the most important and relevant domains to assess and create a more encompassing and multidimensional HR-QoL measurement tool for pediatric CI patients.

## CONCLUSION

To develop a comprehensive assessment tool that accurately measures the HR-QoL of pediatric CI recipients, it is essential to consider the unique functional needs and developmental stages of these patients. Our review highlights the strengths of current assessment tools and the necessity for more detailed evaluations to identify barriers to improved QoL outcomes and prevent their negative impacts. Based on insights from the literature, one key recommendation is the inclusion of age-appropriate and developmentally sensitive domains that capture the evolving challenges faced by CI users. For younger children, domains should emphasize early language acquisition, social engagement in play, and integration into educational settings. In contrast, tools for adolescents should incorporate aspects of independence, self-advocacy, peer relationships, and emotional resilience, as these become more critical during this stage of development. Furthermore, applying rigorous psychometric methodologies, such as item response theory, can ensure that the tools are sensitive to developmental variations and provide meaningful data across a range of abilities. Finally, to address disparities, sociodemographic factors such as race, ethnicity, and socioeconomic status should be considered during the development and validation of these tools, ensuring their applicability and fairness across diverse populations. Given the limited use of CI-specific HR-QoL tools in pediatric populations, it remains challenging to fully understand a child’s ability to communicate consistently and their self-confidence in social interactions.

Our study shows significant differences in QoL outcomes across developmental stages, from childhood to adolescence. The QoL of a preschool-aged child with a CI will likely differ from that of a teenager. Thus, the domains included in a CI-specific HR-QoL tool should be age-appropriate and relevant to the target population to ensure accurate reporting of the real-world impact of CI use. For children, the domains should focus on areas such as speech and language development, social interactions, emotional well-being, and physical health. For adolescents, additional domains such as self-esteem, academic performance, and social integration should be included. By tailoring the domains to the specific developmental and social needs of each age group, we can better capture the overall HR-QoL for pediatric CI users. Quantifying these outcomes will provide valuable insights into the broader pediatric CI population and guide future interventions and support systems. Further investigation is needed to refine these tools and ensure their efficacy across different age groups and developmental stages.

## ACKNOWLEDGMENTS

None declared.

## FUNDING SOURCES

None declared.

## CONFLICT OF INTEREST

M.H. holds the position of Associate Editor for Otology & Neurotology Open and has been recused from reviewing of making decisions for the manuscript. Other author discloses no conflicts of interest.

## Supplementary Material


