# Supplementary material for: The Impact of Cochlear Implantation in Pediatric Patients on Quality of Life: A Systematic Review and Meta-Analysis
Source: Otol Neurotol Open. 2025 Apr 17;5(2):e068. doi: 10.1097/ONO.0000000000000068 (PMC12208647; doi:10.1097/ONO.0000000000000068)
Supplement: Supplementary file 1 [file ono-5-e068-s001.pdf]

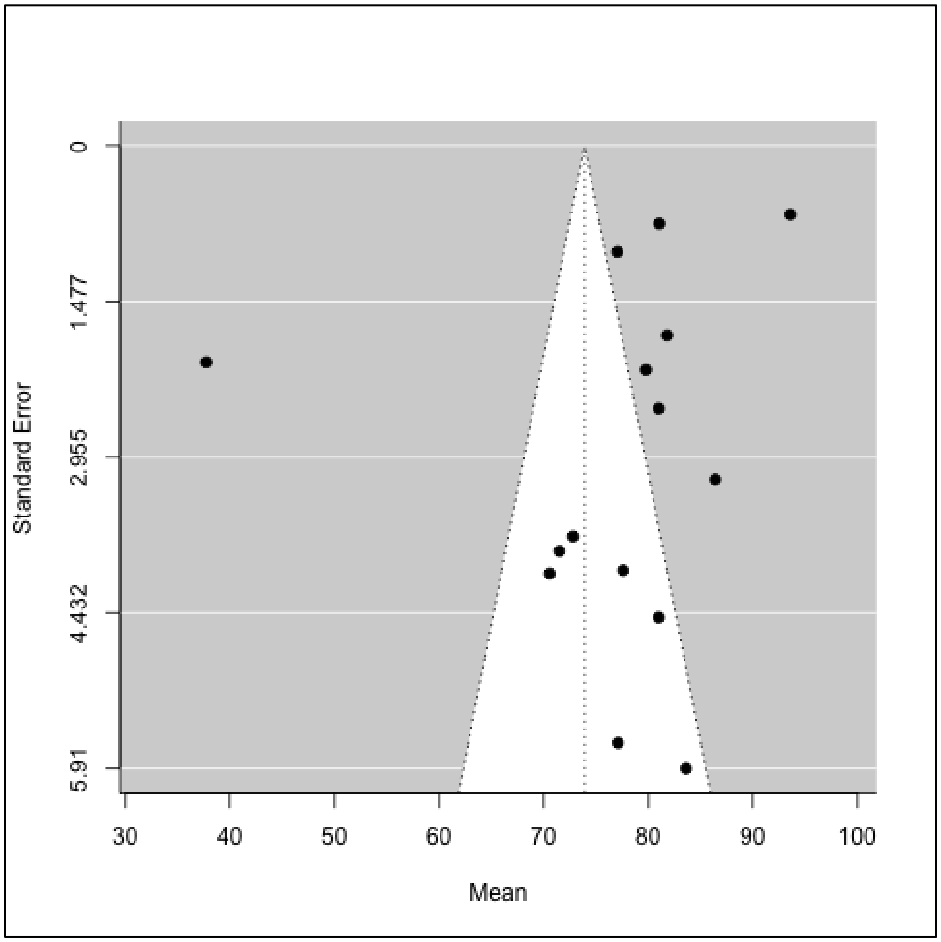

Supplemental Figure 1. Funnel Plot for Physical Well-Being Domain

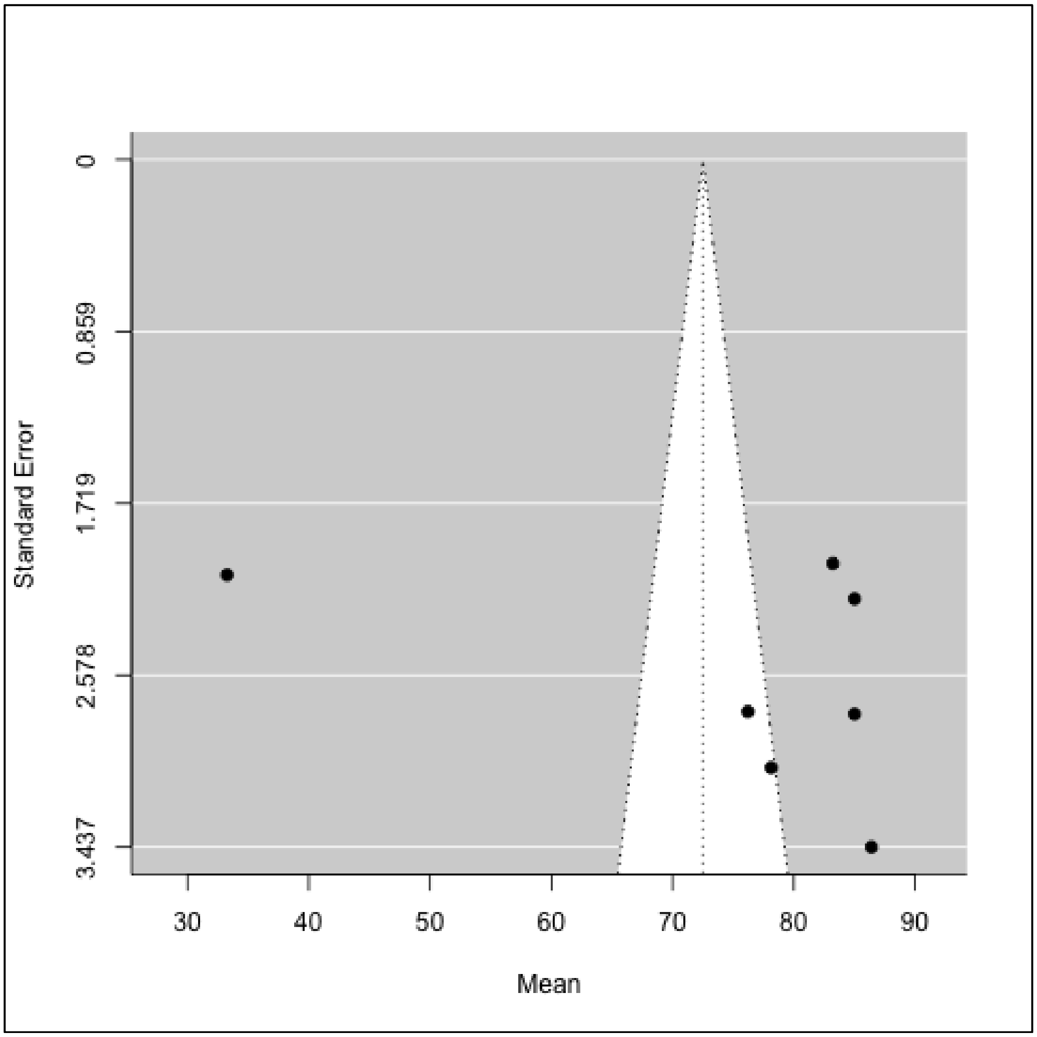

Supplemental Figure 2. Funnel Plot for Psychological Well-Being Domain

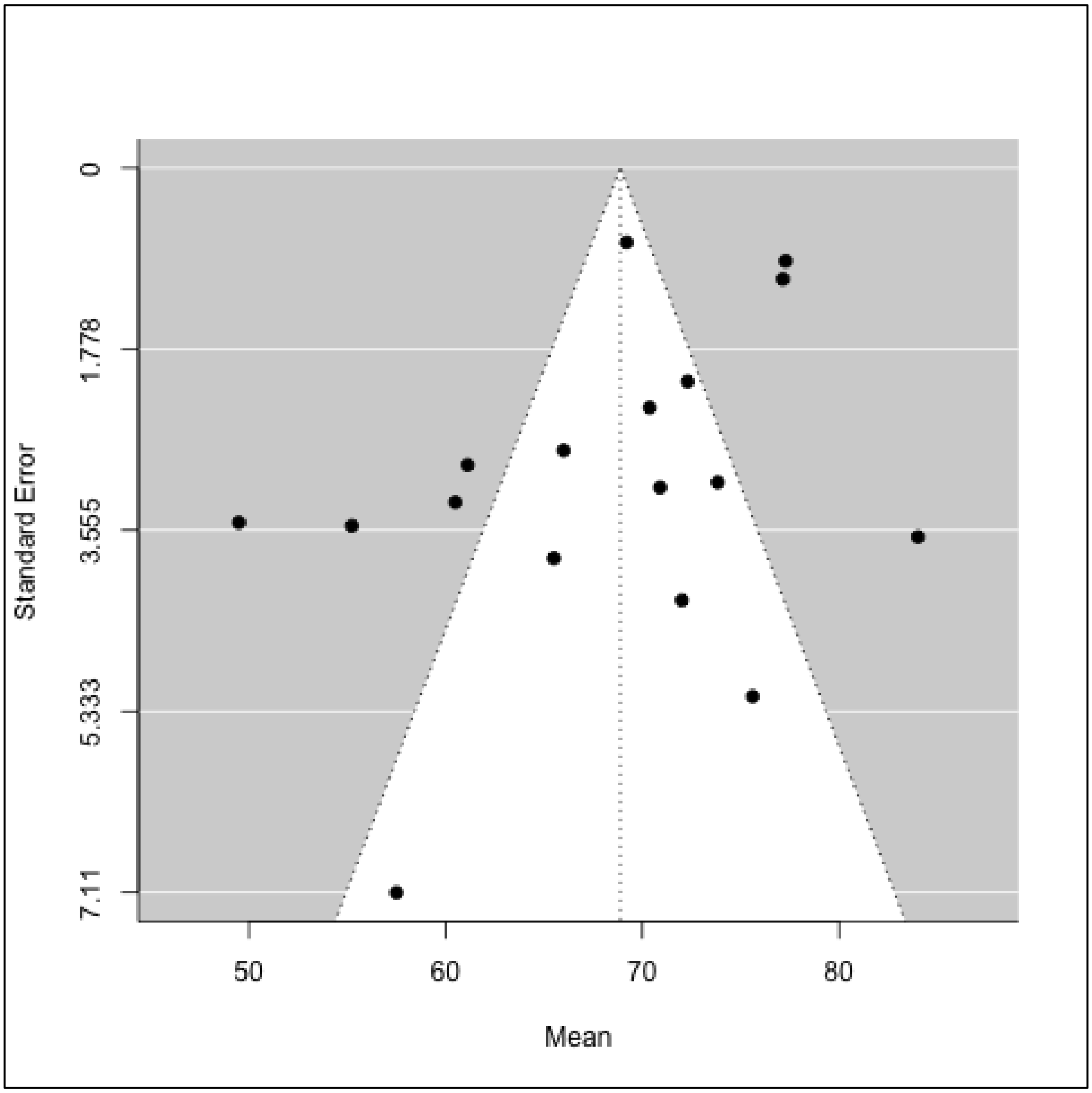

Supplemental Figure 3. Funnel Plot for School Functioning Domain

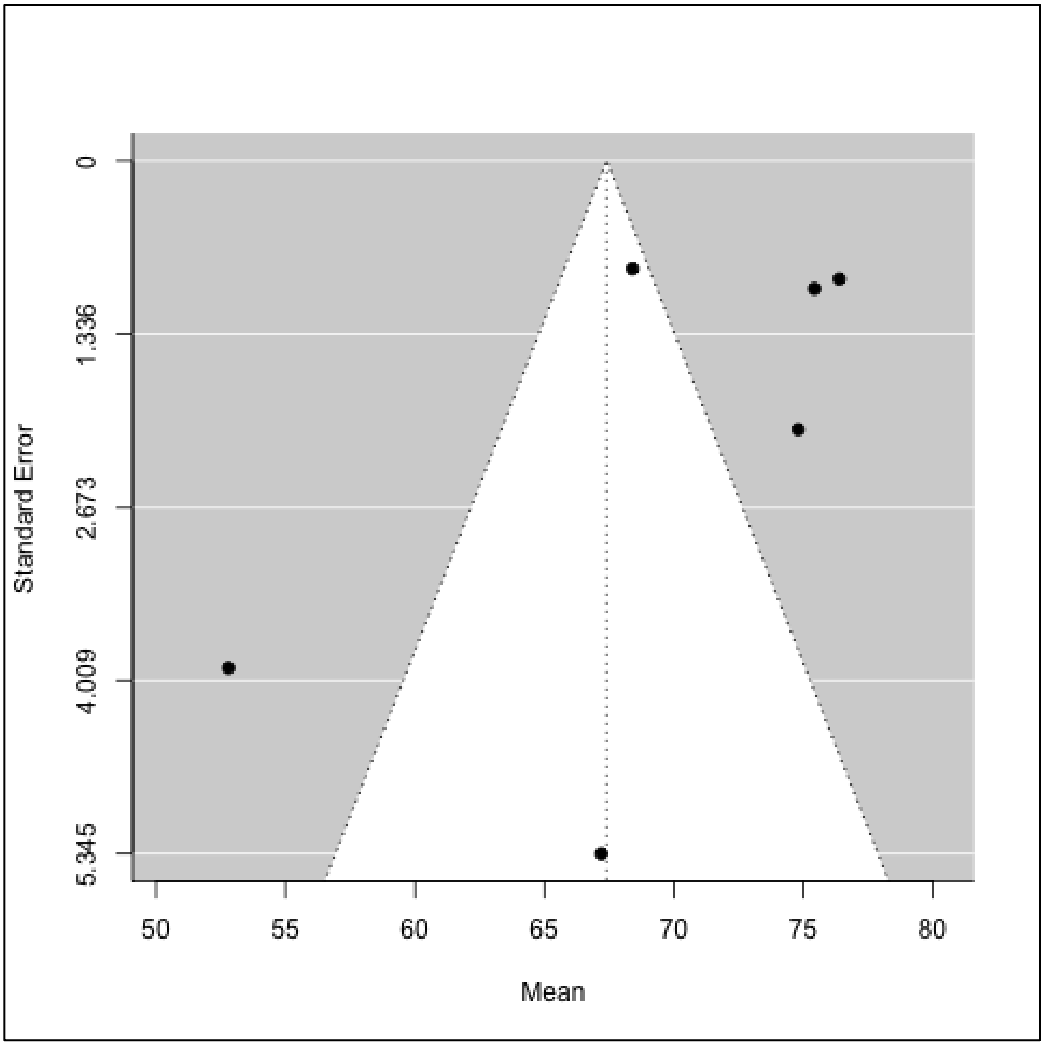

Supplemental Figure 4. Funnel Plot for Emotional Functioning Domain

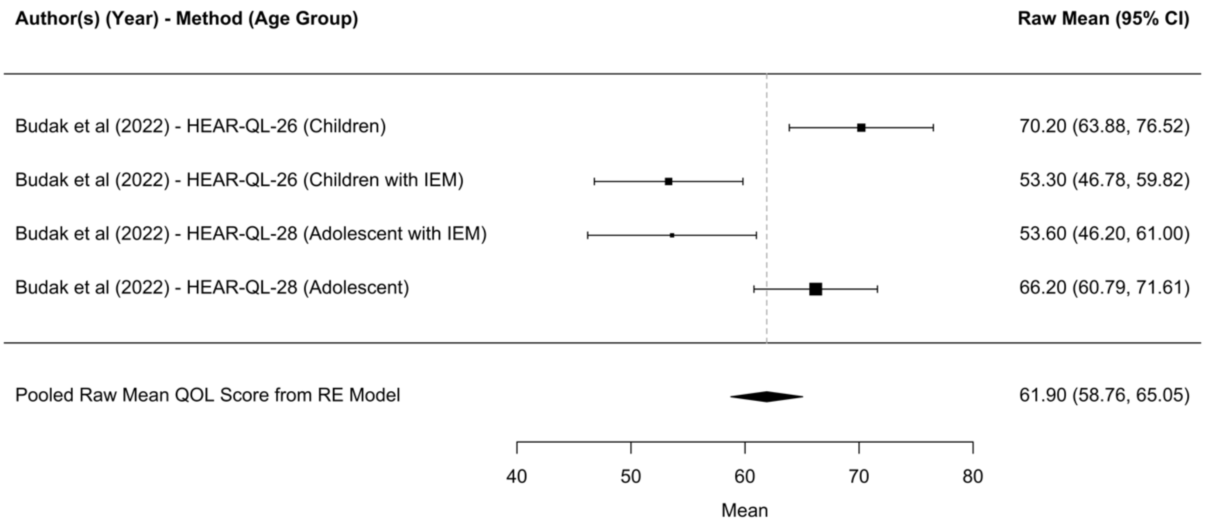

Supplemental Figure 5. Forest Plot for Feelings Domain

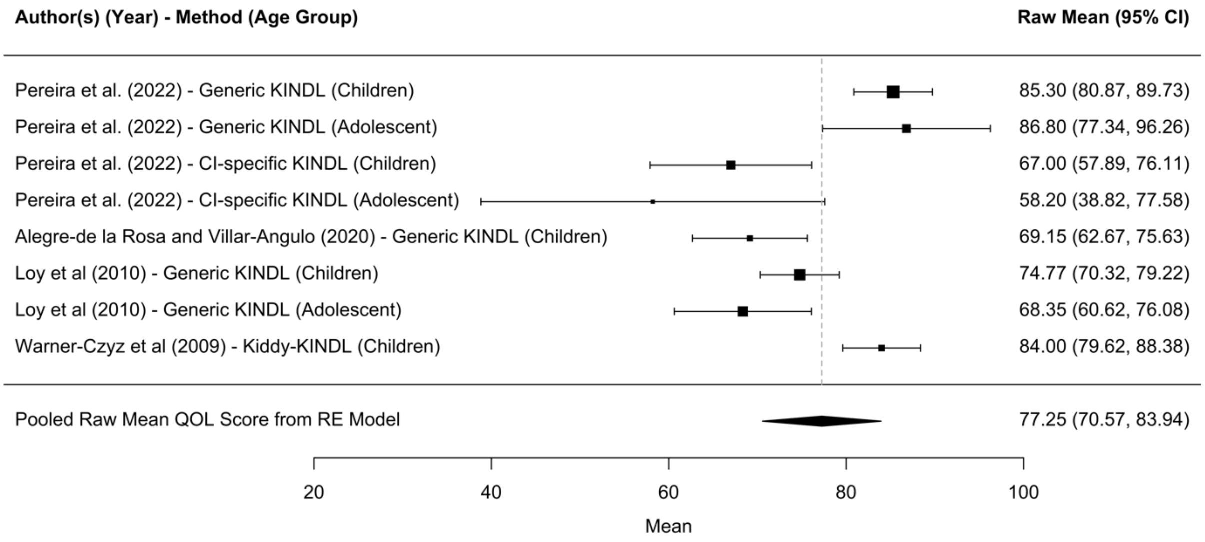

Supplemental Figure 6. Forest Plot for Friends Domain
